# Educators’ perceived mental health literacy and capacity to support students’ mental health: associations with school-level characteristics and provision in England

**DOI:** 10.1093/heapro/daab010

**Published:** 2021-03-01

**Authors:** Rosie Mansfield, Neil Humphrey, Praveetha Patalay

**Affiliations:** 1 Centre for Longitudinal Studies, University College London, WC1E 6BT, UK; 2 Institute of Education, University of Manchester, M13 9PL, UK; 3 MRC Unit for Lifelong Health & Ageing, University College London, WC1E 6BT, UK

**Keywords:** educator, mental health literacy, capacity, school-level predictors

## Abstract

Conceptual frameworks for school-based, preventive interventions recognise that educators’ capacity is, in part, dependent on school-level characteristics. This study aimed to (i) examine the factor structure and internal consistency of the *Mental Health Literacy and Capacity Survey for Educators* (MHLCSE); (ii) assess responses in relation to supporting students’ mental health; (iii) describe schools’ mental health provision in terms of designated roles, training offered, and perceived barriers; (iv) investigate variance in MHLCSE outcomes explained by schools; and, (v) explore school-level predictors of educators’ perceived MHL and capacity after controlling for individual-level characteristics. A multi-level, cross-sectional design involving 710 educators across 248 schools in England was used, and secondary analyses of baseline data collected as part of the Education for Wellbeing Programme were conducted. Mental health provision data was available for 206 schools, of which 95% offered training to some staff, and 71% had a designated mental health lead. Secondary schools offered significantly more training than primary schools. Significant barriers included lack of capacity in Child and Adolescent Mental Health Services (CAMHS) and within school, and communication challenges between agencies. The amount of training offered by schools significantly predicted educators’ awareness and knowledge of mental health issues, treatments and services, legislation and processes for supporting students’ mental health and comfort providing active support, with increased training predicting higher scores. However, little variance was explained by schools (1.7–12.1%) and school-level variables (0.7–1.2%). Results are discussed in relation to current mental health and education policy in England.

## INTRODUCTION

Schools have long been recognised as strategic sites for developing the social and emotional competencies of children and young people, delivering evidence-based mental health interventions, and identifying students at risk (Durlak *et al.*, 2011; Lendrum *et al.*, 2013; Fazel *et al.*, 2014). However, school-based mental health provision varies considerably by country and school type (Patalay *et al.*, 2016). Despite many schools’ commitment to promoting mental health and identifying, supporting and referring students in need, commonly reported barriers to delivering mental health provision include a lack of national policy, guidance and funding and limited staff capacity (Patalay *et al.*, 2016; Department for Education, 2017). To date, school-based mental health provision in England has been more reactive, focused on targeted approaches for students already identified as experiencing difficulties (Patalay *et al.*, 2017). Provision is often not evidence-based, and schools report a lack of consultation and support from external mental health professionals (Vostanis *et al.*, 2013; Sharpe *et al.*, 2016).

A multi-level, multi-sectorial approach to promoting child and adolescent mental health, and improving access to services, is increasingly recognised as an international priority (O’Connell *et al.*, 2009; WHO, 2013; Fazel *et al.*, 2014). In England, recent policy directives have mandated an increased role of schools to promote and protect child and adolescent mental health (Education and Health Committees, 2017). These include statutory guidelines for the introduction of compulsory mental health education by late 2020 (Department for Education, 2019). In addition, it was recommended that all schools appoint a designated mental health lead (Department of Health and Education, 2017). This individual should oversee a whole school approach to mental health and wellbeing and be the identified link for external mental health services. The approaches set out by the government have been heavily criticised for increasing the pressure on schools without the commitment of necessary funding (Education and Health and Social Care Committees, 2018).

From an ecological perspective, teachers are well placed to support children and young people’s mental health (Atkins *et al.*, 2010), and are increasingly undertaking health work (Rossi *et al.*, 2016). When asked whether schools should play a role in supporting the mental health needs of their students, and providing mental health education, 90% of teachers agreed that they should (Graham *et al.*, 2011; Reinke *et al.*, 2011). However, despite 93% of teachers feeling concerned about their students’ mental health, 85% felt they required further mental health training (Moon *et al.*, 2017). More recent qualitative research conducted in the United Kingdom (UK) showed that teachers perceived a lack of clarity in their role and wanted interactive and practical training led by experts (Shelemy *et al.,* 2019a, 2019b). The number of teacher-led mental health education interventions is increasing (Whitley *et al.*, 2013), and for some, educator mental health literacy (MHL) is the key ingredient for improved student outcomes (Kutcher *et al.*, 2015; Miller *et al.*, 2019). However, despite an increased responsibility to implement school-based, mental health programmes, there is limited evidence relating to educators’ reported level of comfort with mental health content, and what training and support is most effective (Whitley *et al*., 2013).

When assessing baseline scores of teachers’ mental health first aid skills, Jorm *et al.* (2010) found low levels of confidence in helping students (<21%), even though over 80% of teachers could correctly recognise depression symptoms. Despite findings from across studies indicating that most teachers can recognise symptoms of mental disorders, awareness of community services and self-reported ability to act on concerns was lacking (Loades and Mastroyannopoulou, 2010). Recent reviews of mental health training programmes for secondary school teachers suggest that more evidence is needed to understand ways to support teachers to improve their helping behaviours and, in turn, students’ mental health outcomes (Booth *et al.*, 2017; Anderson *et al.*, 2019).

Previous research suggests that time constraints are a key barrier to providing help to students (Ekornes, 2017), but that training, clear roles, and support from senior and pastoral teams can help teachers effectively support young people’s mental health and wellbeing (Mazzer and Rickwood, 2015). These findings support conceptual frameworks for school-based, preventive interventions that recognise that teachers’ capacity is, in part, dependent on school-level characteristics and resources, and macro-level factors such as policy and capital (Domitrovich *et al.*, 2008). Individual factors previously found to predict perceived levels of mental health related capabilities include educator gender and year group taught, however, years in practice was not found to be significantly associated (Askell-Williams and Cefai, 2014).

The interaction between individual and school-level factors is highlighted in the multi-level, multi-agency approach adopted in the School Mental Health ASSIST programme in Ontario, Canada (Fortier *et al.*, 2017). The approach presents teachers’ responsibilities as tiered, from the promotion of positive mental health in the classroom, to identification and referral, through to bridging the gap between school support and external agencies. Although teachers may not always be the individual referring a student, awareness and knowledge of the processes for referrals and available interventions can help teachers support these students in the classroom.

Fortier *et al.* (2017) defined MHL as *‘*knowledge, understanding, skill and confidence related to mental health and wellbeing’ in terms of creating mentally healthy classroom environments by reducing stigma, identifying students in need, supporting them through pathways of care and delivering mental health content (p.69). With the aim of revealing which mental health initiatives to prioritise, the *Mental Health Literacy and Capacity Survey for Educators* (MHLCSE) was developed to assess teachers’ awareness, knowledge and comfort relating to supporting students’ mental health. A case study of educators in the Thames Valley school district in Ontario, Canada, revealed lower average scores for items relating to steps for accessing local community support and legislation relating to mental health issues. Fortier *et al.* (2017) did not report on the psychometric properties of the MHLCSE and suggested that more psychometric work is needed to ensure its reliability and validity. In addition, they recommended that links between training provision and capacity should be modelled to better understand what school-level initiatives can help support educators.

There are currently no studies that model both individual and school-level predictors of educators’ perceived capabilities to support students’ mental health. This is important given that implementation of school-based, mental health interventions relies, to an extent, on educators’ capacity. In light of this, the aims of the current study were to (i) examine the factor structure and internal consistency of the MHLCSE; (ii) assess responses in relation to supporting students’ mental health; (iii) describe schools’ mental health provision in terms of designated roles, training offered to staff and perceived barriers to implementation of said provision; (iv) investigate variance in MHLCSE outcomes explained by schools; and, (v) explore school-level predictors of educators’ perceived MHL and capacity to support students’ mental health after controlling for individual-level characteristics.

## METHOD

### Design

The current study conducted secondary analyses on baseline data collected as part of the Department for Education funded, Education for Wellbeing Programme (Hayes *et al.*, 2019a, 2019b). The design of the current study was cross-sectional and multi-level (educators within schools).

### Procedure

School recruitment began in March 2018 across England. The project was advertised via existing school networks, education publications, Public Health England, the National Institute for Health Research, local authorities, school commissioners, and on social media platforms. Schools expressed interest via an online form and provided the name and email address of a self-selected key contact. A survey of mental health provision was completed online by the key contact between July and December 2018. An online survey including the MHLCSE was sent to the staff identified as responsible for the delivery of interventions, if allocated, between September and November 2018 prior to any intervention training.

### Participants

A total of *N* = 710 educators from *N* = 248 schools completed the MHLCSE. The current study was powered to detect effects <0.03 when accounting for the multi-level design. For full power calculations see Supplementary File 1. Educators’ demographic information was only collected in the Education for Wellbeing Programme’s follow up surveys. We therefore extracted this information by using the educator’s unique identifier and merging the data with the baseline MHLCSE responses. Between 24.9% and 35.4% of demographic data were therefore missing from the sample across items due to participant attrition at follow-up. Gender information was available for *N* = 528 (74.4%) educators, of which *N* = 415 (78.6%) identified as female based on these data. Age was reported by *N* = 460 (64.8%) of the sample with a mean age of 37.65 (SD = 9.86), ranging between 22 and 62 years. A total of *N* = 459 (64.6%) educators reported their ethnicity; the majority of the sample were White (*N* = 429, 93.5%). This was slightly higher than the percentage reported in the 2018 school workforce report (91.1%) (Department for Education, 2019). The number of years in practice was reported by *N* = 533 (75.1%) of the sample. On average educators had been practicing for 10.92 (SD = 8.13) years, ranging between 1 and 38 years.

Of the 248 schools for which educator level data was available, a total of 218 key contacts consented to completing a survey of school mental health provision. Twelve schools had completely missing data leaving a total sample of *N* = 206 schools. Of the contacts that completed the survey, *N* = 183 (88.8%) identified as female; the majority were White British (*N* = 193, 93.7%). Just under half were in senior leadership roles (*N* = 100, 48.5%) and a further *N* = 13 (6.3%) were in middle leadership roles. Sixty-eight (33.0%) were in specialist lead roles (e.g. Special Educational Needs Coordinator (SENCO)). Finally, *N* = 14 (6.8%) reported being in a teaching role only, and *N* = 11 (5.3%) were teaching support staff (e.g. teaching assistant). Of the *N* = 206 schools that completed the survey, the majority were mixed sex (*N* = 197, 95.6%), *N* = 131 (63.6%) were primary schools (years 1–6, ages 5–11) and *N* = 75 (36.4%) were secondary schools (years 7–11, ages 12–16).

### Measures

#### Perceived MHL and capacity to support students’ mental health (individual level)

The MHLCSE was designed to have three sub-scales: awareness (items 1–5), knowledge (items 6–9) and comfort (items 10–13). Educators respond using 5-point Likert scales where 1 = *‘*not at all aware*’, ‘*not knowledgeable’ or ‘not comfortable’ and 5 = ‘very aware’, ‘very knowledgeable’ or ‘very comfortable’, respectively*.* The authors of the scale did not conduct any psychometric assessments, and recommend tests of the scale’s reliability and validity prior to use (Fortier *et al.*, 2017).

#### Mental health provision (school level)

The mental health provision survey was developed for the Education for Wellbeing Programme, but was informed by previous research conducted in England (Department for Education, 2017; Day *et al.*, 2018). The current study included variables relating to whether schools had a designated mental health lead (0 = ‘no designated mental health lead’, 1 = ‘designated mental health lead’), which staff were offered training about how to support students’ mental health (two dummy variables created for ‘all teaching staff’ and ‘selected staff only’), what staff training was offered in the last two years relating to students’ mental health (cumulative total score for training provision, 0–84), and the potential barriers to providing effective mental health support within school (total score, 8–32). For full details of items and computation of variables, see Supplementary File 2.

## ANALYSIS STRATEGY

### MHLCSE factor structure

Given that the original authors of the MHLCSE proposed three sub-scales, namely, awareness, knowledge, and comfort, a confirmatory factor analysis (CFA) was conducted first to test the proposed three-factor structure. Next, the factor structure of the MHLCSE was examined by conducting an exploratory factor analysis (EFA) with one to four factors to identify the best structure based on these data. Due to categorical item responses, a robust least squares (WLSMV) estimator was used (Li, 2016). School clustering was accounted for in all models. Good model fit was assessed using the following criteria: an RMSEA value of <0.06 and CFI and TLI values >0.95 (Hu and Bentler, 1999). Due to the ordinal response format, and to ensure better estimates when violating assumptions of tau-equivalence and normality, McDonald’s *ω* was calculated in addition to Cronbach’s α (Trizano-Hermosilla and Alvarado, 2016) when assessing internal consistency of confirmed sub-scales. An average score across items was calculated for each sub-scale identified. All of the above analyses were conducted in Mplus version 8.1.

### Mental health provision

The percentage of schools with a designated mental health lead was calculated and compared across primary and secondary schools. Similarly, the proportion of schools offering training to all teaching staff versus selected staff only was computed. An assessment of the percentage of schools offering training on different topics, by different providers, was conducted and a cumulative training total score was summed to provide a general sense of the level and breadth of opportunity offered to staff to develop their awareness and knowledge. An independent samples *t*-test was used to explore the difference between primary and secondary school training provision. Furthermore, the eight items relating to barriers to providing effective mental health provision were summed to give a total barriers score.

### Missing data analysis

A breakdown of missing data across all variables and complete cases for baseline, individual and full models are included in Supplementary Table 1. Given the amount of missing data for each predictor variable (>5%), complete case analysis was ruled out due to potentially biased estimates and reduced power. Instead, multiple imputation (MI) using chained equations was conducted accounting for school clustering in Stata version 14 prior to running the multi-level models. MI computes multiple predictions for missing values and therefore accounts for uncertainty in imputations, resulting in more accurate standard errors (Azur *et al.*, 2011).

### Multi-level models of individual and school-level predictors of MHLCSE outcomes

Multi-level models for the four MHLCSE outcome variables estimated the proportion of variance explained by schools before including individual and school-level variables. Next, models were fitted including only the individual-level explanatory variables. The final full models fitted both individual and school-level explanatory variables. Model fit was compared across models by comparing –2*log likelihood values, where a lower value indicates better model fit. Coefficients at each level were compared across models, and the proportion of variance explained by adding explanatory variables was calculated. –2*log likelihood and ICC values were computed for each imputed data set and then averaged.

## RESULTS

### MHLCSE factor structure and response distribution

A CFA for three-latent factors (awareness, knowledge and comfort) revealed an inadequate fit (*N* = 710, *χ*^2^ = 896.76; *df* = 62; *p*<0.001; RMSEA [90% CI] = 0.14 [0.13–0.15], CFI = 0.95 TLI = 0.94). All factor loadings were found to be significant with *p*<0.001; however, latent factors were strongly correlated (*r* = 0.66–0.83), and modification indices suggested strong loadings across factors. A clustered EFA with WLSMV estimator was therefore conducted with one to four factors to assess the best structure based on these data (See Supplementary Table 2 for EFA model fit indices.) The EFA revealed that a four-factor structure produced the best model fit. Factor one related to *awareness and knowledge of mental health issues*, factor two *treatments and services*, factor three *legislation and processes for supporting students’ mental health* and factor four, *comfort providing active support* (See Supplementary Table 3 for rotated factor loadings and measures of sub-scale internal consistency, and Supplementary Figure 1 for a model diagram including factor loadings (standard errors), correlations (standard errors) between factors, and residual errors based on the EFA four-factor solution.) All items primarily loaded onto one factor each with the exception of item 13 ‘accessing school and system services for students with mental health issues’, which loaded onto two factors. This item loaded more strongly onto factor four as it was associated with comfort providing active support to students. The findings from the EFA provided further evidence supporting our conclusion from the initial CFA that a three-factor structure did not provide a good fit to our data.

On average, educators reported higher levels of comfort providing active support to students (*M* = 3.52, SD = 0.87), and better awareness and knowledge relating to mental health issues (*M* = 3.61, SD = 0.69) when compared with treatments and services (*M* = 2.98, SD = 0.88) and legislation and processes for supporting students’ mental health (*M* = 3.22, SD = 0.87). See Supplementary Table 4 for the response distribution by item.

### Mental health provision

Out of *N* = 206 schools, *N* = 146 (70.9%) reported that they had a designated mental health lead, with secondary schools proportionately more likely to report this than primary schools (secondary *N* = 59, 78.7%, primary *N* = 87, 66.4%). Of the schools with a designated mental health lead, the following roles and responsibilities were selected: supporting individual students (*N* = 91, 44.2%), teaching students about mental health and wellbeing (*N* = 72, 35.0%), training staff (*N* = 93, 45.1%), liaising with specialist mental health services (*N* = 96, 46.6%) and coordinating and developing mental health provision in the school (*N* = 128, 62.1%). When asked which staff, if any, are offered training about how to support students’ mental health and wellbeing, *N* = 92 (44.7%) schools reported that all teaching staff are offered training. One hundred and three (50.0%) schools reported that only selected members of staff are offered training, and only 11 (5.3%) schools reported that no staff members are offered training. The mean training total was *M* = 8.81 (SD = 7.67) with significantly higher training scores reported by secondary schools (*M* = 10.93, SD = 8.76) than primary schools (*M* = 7.59, SD = 6.71; *t*(204) = –3.08, *p* = 0.002). See Supplementary Table 5 for frequency and percentage of schools offering training across different topics by different providers.

Across all topics, training was most commonly provided by an internal member of staff. Few schools offered training provided online or by higher education institutions. Training relating to recognition of and knowledge relating to risk factors, signs, symptoms and treatments for mental health difficulties was more commonly offered than training relating to legislation and processes for referral and accessing services, and stigma reduction and mental health promotion. Schools reported a wide range of barrier scores (11–32) with an average sum of 21.95 (SD = 3.85) and a mean item score of 2.74 (SD = 0.48) (*N* = 186) (See Supplementary Table 6) The most significant barrier was lack of capacity amongst CAMHS, with almost 80% of schools reporting this as ‘very significant’. Lack of national policy and capacity within school, as well as communication challenges between agencies, were reported as ‘very significant’ barriers by around a third of schools. Few schools (<11%) reported that negative attitudes amongst school staff was a significant barrier to providing effective mental health support.

### Multi-level models of individual and school-level predictors of MHLCSE outcomes

In a multi-level model including eight predictor variables, school type was the only significant school-level predictor of educators’ perceived MHL and capacity (see Supplementary Table 7), with educators from secondary schools reporting higher scores on MHLCSE outcomes. Given the significant relationship between school type and training total score, such that secondary schools offered significantly more training than primary schools, a multi-level model was conducted omitting school type as a predictor to investigate whether total training score significantly predicted educators’ perceived MHL and capacity to support students’ mental health. Table 1 presents the full results of baseline, individual-level and school-level models for all MHLCSE outcome variables when the school type variable is omitted. Little variance was explained by schools (1.7–12.1%) and school-level variables (0.7–1.2%). The only significant school-level predictor of educators’ perceived MHL and capacity was the amount of training offered by schools, with increased training predicting higher MHLCSE scores. A complete case sensitivity analysis was conducted to compare the findings with the multiply imputed models, which produced identical results with respect to significant predictors of MHLCSE outcomes (see Supplementary Table 8).

**Table 1:** Multi-level models for baseline, individual-level predictors only and individual and school-level predictors for MHLCSE outcome (*N* = 710, 248 schools)

	Model 1: Baseline model				Model 2: Individual-level predictors				Model 3: School-level predictors			
Parameter estimate	Estimate (SE)				Estimate (SE)				Estimate (SE)			
	MHI	TS	LP	AS	MHI	TS	LP	AS	MHI	TS	LP	AS
*Educator level*												
Intercept	3.61 (0.03)**	2.98 (0.03)**	3.23 (0.04)**	3.52 (0.04)**	3.46 (0.07)**	2.72 (0.09)**	3.08 (0.09)**	3.43 (0.09)**	3.31 (0.24)**	2.97 (0.33)**	2.90 (0.31)**	3.24 (0.32)**
Gender (female)					0.14 (0.07)	0.12 (0.09)	0.11 (0.09)	0.11 (0.09)	0.12 (0.07)	0.09 (0.09)	0.08 (0.10)	0.09 (0.10)
Years in practice					0.00 (0.00)	0.01 (0.01)*	0.01 (0.00)	0.00 (0.00)	0.00 (0.00)	0.02 (0.01)*	0.01 (0.00)	0.00 (0.00)
*School level*												
Designated MH lead (yes)									0.03 (0.07)	0.00 (0.09)	0.05 (0.09)	0.01 (0.09)
Training: selected staff only (yes)									–0.06 (0.14)	–0.16 (0.19)	–0.05 (0.19)	–0.07 (0.19)
Training: all teaching staff (yes)									0.04 (0.15)	–0.06 (0.19)	–0.01 (0.20)	–0.03 (0.20)
Mean training total									0.01 (0.00)*	0.01 (0.00)*	0.02 (0.01)*	0.01 (0.01)*
Mean barriers total									0.00 (0.01)	–0.01 (0.01)	0.00 (0.01)	0.01 (0.01)
Log-likelihood	–739.80	–912.53	–906.05	–901.42	–735.54	–902.93	–903.47	–900.09	–728.30	–895.04	–895.18	–895.31
ICC	0.017	0.045	0.121	0.110	0.024	0.053	0.128	0.114	0.008	0.038	0.112	0.098
[95% CI]	[0.000–0.491]	[0.009–0.203]	[0.060–0.230]	[0.052–0.217]	[0.001–0.331]	[0.012–0.198]	[0.065–0.237]	[0.055–0.222]	[0.000–0.926]	[0.005–0.229]	[0.052–0.226]	[0.042–0.215]
Random Effects	0.09 (0.09)	0.19 (0.08)	0.30 (0.06)	0.29 (0.05)	0.11 (0.08)	0.20 (0.07)	0.31 (0.05)	0.29 (0.05)	0.03 (0.16)	0.17 (0.08)	0.29 (0.06)	0.27 (0.06)

* *Note.* Sub-scales: MHI, awareness and knowledge of mental health issues; TS, treatments and services; LP, legislation and processes; AS, comfort providing active support. *p*<0.05, **p<0.001.

## DISCUSSION

In order to identify the gaps in educators’ perceived MHL and capacity to support students’ mental health, the MHLCSE was used in the current study. The original three-factor structure, including awareness, knowledge and comfort sub-scales, was not confirmed (Fortier *et al.*, 2017). Instead, an EFA revealed a four-factor structure had the best fit to the data, including factors relating to *awareness and knowledge of mental health issues, treatments and services, legislation and processes* and *comfort providing active support.* These findings suggest that awareness and knowledge are not separate constructs, but rather sub-scales are differentiated by the topics of perceived awareness and knowledge. The four items that made up the original comfort scale remained as one factor; though in the current study this sub-scale was renamed to capture the behavioural element of providing active support. All sub-scales were found to have high levels of internal consistency, providing further support for the four-factor structure.

Educators reported less awareness and knowledge of available treatments and services, and legislation and processes relating to supporting students’ mental health, when compared with awareness and knowledge of risk factors, signs and symptoms and the range of mental health issues experienced by children and young people. This supports previous research that found despite the ability to recognise symptoms of mental disorders, teachers wanted more information relating to community services and processes for acting on their concerns about a student (Loades and Mastroyannopoulou, 2010). Although the current study reports relatively high sub-scale scores for comfort providing active support to students, at an item level, responses support previous literature in identifying many educators who do not feel comfortable with this role (Jorm *et al.*, 2010). Specifically, educators were less confident talking with parents about students’ mental health.

When considering Fortier *et al.*’s (2017) tiered model of educators’ role in supporting students’ mental health, improving awareness and knowledge of treatments and services, as well as processes for referrals, could help educators be more understanding of students in their class who may be accessing treatments, and to support students when bridging the gap between school support and external agencies. Similarly, improving educators’ awareness and knowledge of relevant legislation such as confidentiality and children and young people’s rights, should help to encourage mentally healthy classrooms in which students feel safe to talk about their mental health, and could help improve educators’ comfort when discussing students’ mental health with parents.

In relation to existing provision, over 70% of schools in the current study reported having a designated mental health lead. Compared with previous studies in the UK that showed that approximately 50% of schools identified a designated mental health lead (Department for Education, 2017), this finding could indicate an increased priority afforded to mental health in recent years. This is understandable given the recent introduction of policy recommendations that incentivise schools to identify a mental health lead to oversee provision. However, it must be acknowledged that the schools surveyed in the current study were part of a wider programme of research evaluating a range of school-based mental health interventions. The sample is therefore likely biased towards schools that were particularly motivated to improve their mental health provision, and felt able to commit time to the project. Furthermore, the responsibilities of the designated mental health leads identified in the current study were varied. This could be one reason why this variable was not a predictor of educators’ MHL and capacity in the current study. As a new policy recommendation, these findings suggest that clarification of the roles and responsibilities of school-based, designated mental health leads in England is needed.

The majority of schools reported offering mental health training to some staff within the school, and there was an almost equal split between schools that reported offering training to all teaching staff versus selected staff only. Most training was being delivered by internal members of staff. The majority of schools identified a designated mental health lead and almost half reported that they trained other staff. This could be due to significant barriers reported by schools such as lack of capacity amongst CAMHS, as well as communication challenges between agencies. Lack of capacity within school was also reported as a significant barrier to providing effective mental health support by approximately a third of schools. However, on the whole, schools did not report negative attitudes amongst school staff. These results align with significant barriers reported by schools in earlier research (Day *et al.*, 2018).

In line with gaps in educators’ awareness and knowledge relating to treatments, services, legislation and processes, the need for more training provided by external agencies is evident. These findings support previous research in suggesting that schools generally accept their role in promoting mental health and identifying, supporting and referring students in need (Department for Education, 2017), but lack national policy and guidance, staff capacity and consultation and support from external mental health professionals (Vostanis *et al.*, 2013; Patalay *et al.*, 2016; Sharpe *et al.*, 2016). It must be recognised that schools are being expected to hold greater responsibility in supporting their students’ mental health within the context of an extended period of austerity (Hanley, Winter, & Burrell, 2020). Similarly, many years of underfunding have resulted in insufficient capacity within CAMHS services to deal with increased demand. The future of mental health promotion in schools therefore depends on increased resources, and a public health approach that builds capacity for multi-agency working (Cortina, 2020). An example of a project working to develop better links between sectors is the Mental Health Services and Schools and Colleges Link Programme currently being rolled out in England (Cortina *et al.*, 2019). The Link Programme connects school mental health leads with a key contact within their local NHS children and young people’s mental health service, and offers workshops to develop communication and facilitate joint working.

In terms of what type of training was offered by schools in the current study, the most common topics related to recognition of and knowledge relating to risk factors, signs, symptoms, and treatments for mental health difficulties. Less common topics included legislation and processes for referral and accessing services as well as stigma reduction and mental health promotion. It has been previously found that plans and policies for promoting positive mental health of students are less common than plans to support pupils with identified mental health difficulties (Department for Education, 2017). With the introduction of compulsory mental health education in England, more training is needed that focuses on prevention and promotion as well as the referral process within the local context.

Overall, results of the multi-level models indicate that, before including individual and school-level predictors, schools explained a small amount of variance (<5%) in awareness and knowledge relating to mental health issues, and treatments and services, and relatively larger proportion of variance (11–12%) in awareness and knowledge relating to legislation and processes, and comfort providing active support. Despite slightly improving overall model fit, the addition of individual-level variables in all models did not explain additional variance in MHLCSE outcomes. Gender was not found to significantly predict educators’ MHL and capacity. This finding does not support previous research (Askell-Williams and Cefai, 2014); however, in line with findings from this research, the current study found that, for the majority of MHLCSE outcomes, years in practice was not a significant predictor. Years in practice was however significantly and positively associated with awareness and knowledge relating to treatments and services. It is worth noting that awareness and knowledge of treatments and services differs from capabilities for mental health promotion, as measured in Askell-Williams and Cefai’s (2014) study. This finding could highlight the changing role of educators such that years in practice could have helped accumulate awareness and knowledge of treatments and services, but not confidence providing active support as is increasingly expected of school staff.

Secondary schools were proportionately more likely to report having a designated mental health lead and showed significantly higher levels of training provision when compared with primary schools. Furthermore, educators from secondary schools reported significantly higher levels of MHL and capacity to support students’ mental health. This aligns with previous literature that showed secondary schools have higher levels of mental health provision (Patalay *et al.*, 2017). Despite no direct effect of reporting a designated mental health lead on MHLCSE outcomes, the increased likelihood of secondary schools to report this role may be indirect support for clear roles and support from senior and pastoral teams helping teachers effectively support young people’s mental health (Mazzer and Rickwood, 2015). Higher levels of mental health provision in secondary schools makes sense given that they are larger, and due to the age of onset of many mental health difficulties, are likely to have a higher proportion of students developing mental health difficulties (Kim-Cohen *et al.*, 2003; WHO, 2013). However, this supports a more reactive approach, focused on providing targeted support. With greater responsibility to promote positive mental health, and prevent experiences of mental distress in adolescence, greater attention should be given to improving primary school provision.

In order to explore the effect of the total training score on educators’ MHL and capacity, beyond that explained by school type, models were run excluding school type as a predictor variable. The final models, including both individual and school-level variables, explained additional variance in educators’ perceived MHL and capacity. However, only training total was found to be a significant predictor across all MHLCSE outcomes, with higher training total scores at the school level predicting greater levels of educators’ perceived MHL and capacity to support students’ mental health. These findings support universal approaches to school-based interventions that understand that educators’ capacity is dependent, in part, on school-level characteristics and resources (Domitrovich *et al.*, 2008). However, the variance in MHLCSE outcomes explained by school-level training provision was small, and perceived school-level barriers to providing effective mental health support did not significantly predict MHLCSE outcomes. Overall, the findings from the multi-level models indicated that a relatively small amount of variance in MHLCSE outcomes was explained by differences between schools and their characteristics.

## LIMITATIONS

Secondary analyses were conducted using cross-sectional data. Conclusions must therefore be drawn with caution about the influence of school-level characteristics on educators’ perceived MHL and capacity, given that it was not possible to determine whether mental health provision reported at the school-level had actually been *experienced* by the educators completing the MHLCSE. Instead, the school-level variables offered a more general sense of the spread of responsibility of school staff to support students’ mental health, and the level of opportunity offered to staff for mental health related training. Furthermore, the contacts responsible for completing the mental health provision survey, worked across a number of different roles. It is therefore worth considering the possible influence of role on the mental health provision reported. Similarly, the role of the educators could have explained some of the variance in MHLCSE outcomes (e.g. being a classroom teacher and the SENCO), and this is therefore a limitation of the study. Educators’ direct and indirect experience of mental health difficulties could also have contributed to higher levels of MHL (Ten Have *et al.*, 2010). Future research should therefore account for these individual differences to better understand within school variations in MHL and capacity.

Despite being powered to detect small effects, the secondary nature of this study also meant that the average cluster size was relatively small. A larger number of educators per school could have resulted in more precise estimates of school-level variance (ICCs). With the merging of school and individual-level surveys in the current study, there was missing data where one of the data sources was incomplete. For example, although educators from across 248 schools completed the MHLCSE, not all of these schools’ key contacts completed the mental health provision survey. Furthermore, demographic information was not available for all educators that completed the MHLCSE. This issue of data completeness might have biased our estimates in the current study. However, this is counterbalanced by our use of imputation methods and complete case sensitivity analyses to assess the influence of imputation on the results. The study therefore provided complete transparency in terms of the amount of missing data, its treatment and the impact on outcomes.

## CONCLUSION

In the current study, educators reported lower awareness and knowledge relating to legislation and processes for accessing community services. This appears to be a particularly important area for development along with supporting educators to feel more confident talking with parents about students’ mental health. The majority of schools had a designated mental health lead and at least some selected staff members were being offered mental health training. However, training relating to legislation and processes for supporting students’ mental health, stigma reduction and mental health promotion were less commonly offered, particularly by external organisations. Secondary schools were more likely to have a designated mental health lead and higher levels of training provision. More work is therefore needed to improve primary school mental health provision. Higher levels of training offered at the school-level was associated with increased educator MHL and capacity. However, having a designated mental health lead, offering training to all teaching staff vs. selected members of staff and school-level barriers to providing effective support did not significantly predict MHLCSE in the current study, and relatively little variance in outcomes was explained by schools and school-level characteristics. More research is needed to fully understand the meaning of these results and the true implications for educator mental health training.

## SUPPLEMENTARY MATERIAL

Supplementary material is available at *Health Promotion International* online.

## FUNDING

This work (the Education for Wellbeing Programme and RMs PhD) was supported by the Department for Education, England [grant number: EOR/SBU/2017/015].

## CONFLICT OF INTEREST STATEMENT

The views expressed are those of the authors and not necessarily those of the Department for Education, England or its arm’s length bodies, or other Government Departments. The authors declare no conflict of interest and take sole responsibility for the content of this article.

## ETHICS APPROVAL

Approval was obtained from University College London Research Ethics Committee [6735/009, 6735/014].

## Supplementary Material

daab010_Supplementary_Data
