# Supplementary material for: Educators’ perceived mental health literacy and capacity to support students’ mental health: associations with school-level characteristics and provision in England
Source: Health Promot Int. 2021 Mar 1;36(6):1621–32. doi: 10.1093/heapro/daab010 (PMC8699399; doi:10.1093/heapro/daab010)
Supplement: daab010_Supplementary_Data [file daab010_supplementary_data.zip › RM_rpSupplementary Tables Clean Version..docx]

**Supplementary Tables**

Supplementary Table 1. *Breakdown of missing data across variables and complete cases for baseline, individual level and full models including both individual and school-level predictors, number of schools and average cluster size*

| Models and Predictor Variables | Missing Data | Complete Cases | Number of Schools | Average Cluster Size |
| --- | --- | --- | --- | --- |
| Baseline Models | 0(0.0) | 710(100.0) | 248 | 2.86 |
| Individual-level Models | 182(25.6) | 528(74.4) | 230 | 2.30 |
| Gender | 182(25.6) | 528(74.4) | - | - |
| Years in Practice | 177(24.9) | 533(75.1) | - | - |
| Full Models | 294(41.4) | 416(58.6) | 175 | 2.38 |
| Designated MH Lead | 119(16.8) | 591(83.2) | - | - |
| Training: selected staff only | 123(17.3) | 587(82.7) | - | - |
| Training: all teaching staff | 123(17.3) | 587(82.7) | - | - |
| Mean Training Total | 123(17.3) | 587(82.7) | - | - |
| Mean Barriers Total | 170(23.9) | 540(76.1) | - | - |

*Note.* Missing data and complete cases presented as count(%)

Supplementary Table 2. *Model fit indices for one to four factor solutions based on the clustered EFA using WLSMV estimator (N = 710)*

| Model | χ^2^(df) | RMSEA [90% CI] | CFI | TLI |
| --- | --- | --- | --- | --- |
| One-factor | 1943.38(65)** | .20[.19-.21] | .90 | .88 |
| Two-factor | 1076.27(53)** | .17[.16-.17] | .94 | .92 |
| Three-factor | 700.56(42)** | .15[.14-.16] | .96 | .93 |
| Four-factor | 216.67(32)** | .09[.08-.10] | .99 | .98 |

*Note*. **p<.001.

Supplementary Figure 1. *The Four-Factor Structure for the MHLCSE based on the clustered EFA using WLSMV estimator*

[see Supplementary Figure 1.jpg]

*Note.* The model includes factor loadings (standard errors), correlations (standard errors) between factors and residual errors; * p<.05.

Supplementary Table 3. *Rotated factor loadings for a four-factor solution (based on the clustered EFA using WLSMV estimator) along with measures of sub-scale internal consistency (N = 710)*

| Item | 1  Awareness and knowledge of MHI | 2  Treatments and Services | 3  Legislation and Processes | 4  Comfort Providing Active Support |
| --- | --- | --- | --- | --- |
| 1. The range of mental health issues that children and youth experience during the school years. | .81* |  |  |  |
| 1. The risk factors and causes of student mental health issues. | .81* |  |  |  |
| 1. The types of treatments available to help students with mental health issues (e.g. counselling). |  | .44* |  |  |
| 1. The local community services for treating students with mental health issues (e.g. do you know who to call?). |  | .97* |  |  |
| 1. The steps necessary to access local community services for mental health issues. |  | .76* |  |  |
| 1. About the signs and symptoms of student mental health issues. | .50* |  |  |  |
| 1. About appropriate actions to take to support student mental health at school. |  |  | .71* |  |
| 1. About legislation related to mental health issues (confidentiality, consent to treatment, etc.). |  |  | .67* |  |
| 1. About school system services and resources for helping students with mental health issues. |  |  | .84* |  |
| 1. Talking with students about mental health. |  |  |  | .88* |
| 1. Talking with parents about their child’s mental health. |  |  |  | .70* |
| 1. Providing support to students with mental health issues. |  |  |  | .78* |
| 1. Accessing school and system services for students with mental health issues. |  |  |  | .55* |
| Cronbach’s alpha (α) | .89 | .89 | .88 | .91 |
| McDonald’s omega (*ω*) | .89 | .90 | .88 | .92 |

*Note*. * p<.05.

Supplementary Table 4. *Item response distributions and average sub-scale scores on the Mental Health Literacy and Capacity Survey for Educators (N = 710)*

| Item | 1 (not at all) | 2 | | 3 | | 4 | | 5 (very) |
| --- | --- | --- | --- | --- | --- | --- | --- | --- |
| 1. The range of mental health issues that children and youth experience during the school years. | 3(0.4) | 16(2.3) | | 272(38.3) | | 297(41.8) | | 122(17.2) |
| 1. The risk factors and causes of student mental health issues. | 1(0.1) | 37(5.2) | | 264(37.2) | | 307(43.2) | | 101(14.2) |
| 1. The types of treatments available to help students with mental health issues (e.g. counselling). | 8(1.1) | 108(15.2) | | 314(44.2) | | 212(29.9) | | 68(9.6) |
| 1. The local community services for treating students with mental health issues (e.g. do you know who to call?). | 55(7.7) | 205(28.9) | | 264(37.2) | | 135(19.0) | | 51(7.2) |
| 1. The steps necessary to access local community services for mental health issues. | 79(11.1) | 223(31.4) | | 261(36.8) | | 102(14.4) | | 45(6.3) |
| 1. About the signs and symptoms of student mental health issues. | 5(0.7) | 62(8.7) | | 316(44.5) | | 268)37.7) | | 59(8.3) |
| 1. About appropriate actions to take to support student mental health at school. | 11(1.5) | 84(11.8) | | 266(37.5) | | 261(36.8) | | 88(12.4) |
| 1. About legislation related to mental health issues (confidentiality, consent to treatment, etc.). | 70(9.9) | 171(24.1) | | 258(36.3) | | 156(22.0) | | 55(7.7) |
| 1. About school system services and resources for helping students with mental health issues. | 26(3.7) | 131(18.5) | | 263(37.0) | | 207(29.2) | | 83(11.7) |
| 1. Talking with students about mental health. | 11(1.5) | 37(5.2) | | 189(26.6) | | 263(37.0) | | 210(29.6) |
| 1. Talking with parents about their child’s mental health. | 28(3.9) | 134(18.9) | | 256(36.1) | | 201(28.3) | | 91(12.8) |
| 1. Providing support to students with mental health issues. | 18(2.5) | 92(13.0) | | 233(32.8) | | 232(32.7) | | 135(19.0) |
| 1. Accessing school and system services for students with mental health issues. | 17(2.4) | 119(16.8) | | 249(35.1) | | 207(29.2) | | 118(16.6) |
| Sub-scale | MHI | | TS | | LP | | AS | |
| Possible range of scores | (3-15) | | (3-15) | | (3-15) | | (4-20) | |
| Total sum, minimum – maximum, mean(±SD) | 4-15, 10.84(±2.06) | | 3-15, 8.94(±2.63) | | 3-15, 9.67(±2.62) | | 4-20, 14.09(±3.47) | |
| Average, minimum – maximum, mean(±SD) | 1-5,  3.61(±.69) | | 1-5,  2.98(±.88) | | 1-5,  3.22(±.87) | | 1-5,  3.52(±.87) | |

*Note.* item response distributions are presented as count(%), 1 = not at all aware, knowledgeable or comfortable; 5 = very aware, knowledgeable or comfortable, sub-scales: MHI – awareness and knowledge of mental health issues; TS = treatments and services; LP = legislation and processes; AS = comfort providing active support.

Supplementary Table 5. *Frequency and percentage of schools offering training across different topics by different providers (N = 206)*

|  | Provided by  a member of  staff within  the school  (e.g. mental  health lead, SENCO) | Provided by  a higher  education  institution  (e.g. University) | Provided by  local NHS  Child and  Adolescent  Mental Health  Services (CAMHS) | Provided by  a voluntary organisation | Provided by  an  independent contractor | Provided by  local authority | Online course (e.g. MindEd) |
| --- | --- | --- | --- | --- | --- | --- | --- |
| Understanding the range of mental health difficulties that children and youth experience during the school years | 73(35.4) | 3(1.5) | 36(17.5) | 26(12.6) | 27(13.1) | 37(18.0) | 15(7.3) |
| Signs and symptoms of student mental health difficulties | 67(32.5) | 3(1.5) | 33(16.0) | 24(11.7) | 26(12.6) | 37(18.0) | 15(7.3) |
| How to identify mental health needs among pupils and recognise specific mental health difficulties | 56(27.2) | 4(1.9) | 26(12.6) | 22(10.7) | 23(11.2) | 30(14.6) | 10(4.9) |
| Risk factors and causes of student mental health difficulties | 61(29.6) | 3(1.5) | 27(13.1) | 16(7.8) | 28(13.6) | 33(16.0) | 11(5.3) |
| The types of interventions available to help students with mental health difficulties | 67(32.5) | 3(1.5) | 34(16.5) | 15(7.3) | 22(10.7) | 41(19.9) | 9(4.4) |
| Legislation related to young peoples’ mental health difficulties (confidentiality, consent to treatment, etc.) | 31(15.0) | 5(2.4) | 9(4.4) | 8(3.9) | 15(7.3) | 20(9.7) | 7(3.4) |
| Understanding school systems and resources for students with mental health difficulties | 93(45.1) | 0(0.0) | 9(4.4) | 10(4.9) | 13(6.3) | 22(10.7) | 3(1.5) |
| Understanding local community services for students with mental health difficulties | 40(19.4) | 0(0.0) | 20(9.7) | 10(4.9) | 7(3.4) | 32(15.5) | 4(1.9) |
| Understanding appropriate referral actions and steps to accessing local community services | 62(30.1) | 0(0.0) | 21(10.2) | 6(2.9) | 10(4.9) | 28(13.6) | 3(1.5) |
| Mental health first aid | 28(13.6) | 0(0.0) | 15(7.3) | 18(8.7) | 23(11.2) | 33(16.0) | 6(2.9) |
| Stigma awareness and promoting stigma reduction | 64(31.1) | 1(0.5) | 7(3.4) | 16(7.8) | 12(5.8) | 16(7.8) | 6(2.9) |
| Knowledge of how to obtain and maintain mental health | 58(28.2) | 0(0.0) | 10(4.9) | 16(7.8) | 11(5.3) | 18(8.7) | 5(2.4) |
| None | 23(11.2) | 22(10.7) | 18(8.7) | 22(10.7) | 20(9.7) | 19(9.2) | 20(9.7) |

*Note.* results are presented as count(%)

Supplementary Table 6. *Item response distributions and descriptive statistics for items relating to barriers to providing effective school mental health support*

|  | Not at all significant/don’t know | Not very significant | Quite significant | Very significant |
| --- | --- | --- | --- | --- |
| Lack of information about locally available support for mental health issues (N = 201) | 12(6.0) | 45(22.4) | 96(47.8) | 48(23.9) |
| Poor communication between different agencies (N = 196) | 13(6.6) | 26(13.3) | 94(48.0) | 63(32.1) |
| Lack of national policy for mental health in schools (N = 202) | 12(5.9) | 34(16.8) | 88(43.6) | 68(33.7) |
| Low priority afforded to mental health within the school inspection regime (N = 199) | 31(15.6) | 60(30.2) | 60(30.2) | 48(24.1) |
| Negative attitudes towards mental health amongst staff in my school (N = 195) | 122(62.6) | 52(26.7) | 16(8.2) | 5(2.6) |
| Lack of capacity within my school (e.g. time, availability, training) (N = 201) | 13(6.5) | 35(17.4) | 90(44.8) | 63(31.3) |
| Recruitment and retention difficulties with specialist staff in my school (N = 196) | 78(39.8) | 56(28.6) | 38(19.4) | 24(12.2) |
| Lack of capacity amongst NHS Child and Adolescent Mental Health Services (CAMHS) (N = 198) | 4(2.0) | 6(3.0) | 31(15.7) | 157(79.3) |
| Total Sum (4-32), minimum–maximum, mean(±SD) (N = 186) | 11-32,  21.95(±3.85) | | | |
| Average (1-4), minimum–maximum, mean(±SD) (N = 186) | 1-4,  2.74(±.48) | | | |

*Note:* item response distributions are presented as count(%)

Supplementary Table 7. *Multi-level models for baseline, individual-level predictors only and individual and school-level predictors for MHLCSE outcome – including school type as a predictor (N = 710, 248 schools)*

|  | Model 1: Baseline Model | | | | | | | | Model 2: Individual-level Predictors | | | | | | | | Model 3: School-level Predictors | | | | | | |
| --- | --- | --- | --- | --- | --- | --- | --- | --- | --- | --- | --- | --- | --- | --- | --- | --- | --- | --- | --- | --- | --- | --- | --- |
| Parameter Estimate | Estimate(SE) | | | | | | | | Estimate(SE) | | | | | | | | Estimate(SE) | | | | | | |
|  | MHI | | TS | | LP | | AS | | MHI | | TS | | LP | | AS | | MHI | | TS | | LP | | AS |
| Educator-level |  | |  | |  | |  | |  | |  | |  | |  | |  | |  | |  | |  |
| Intercept | 3.61(.03)** | | 2.98(.03)** | | 3.23(.04)** | | 3.52(.04)** | | 3.46(.07)** | | 2.72(.09)** | | 3.08(.09)** | | 3.43(.09)** | | 3.20(.23)** | | 2.83(.32)** | | 2.75(.30)** | | 3.13(.32)** |
| Gender (female) |  | |  | |  | |  | | .14(.07) | | .12(.09) | | .11(.09) | | .11(.09) | | .14(.07) | | .12(.09) | | .11(.10) | | .12(.10) |
| Years in Practice |  | |  | |  | |  | | .00(.00) | | .01(.01)* | | .01(.00) | | .00(.00) | | .00(.00) | | .02(.00)* | | .01(.00) | | .00(.00) |
| School-level |  | |  | |  | |  | |  | |  | |  | |  | |  | |  | |  | |  |
| School Type (secondary) |  | |  | |  | |  | |  | |  | |  | |  | | .25(.05)** | | .33(.07)** | | .40(.07)** | | .28(.08)** |
| Designated MH Lead (yes) |  | |  | |  | |  | |  | |  | |  | |  | | .01(.07) | | -.02(.08) | | .01(.09) | | -.01(.09) |
| Training: selected staff only (yes) |  | |  | |  | |  | |  | |  | |  | |  | | -.10(.14) | | -.21(.18) | | -.11(.18) | | -.12(.18) |
| Training: all teaching staff (yes) |  | |  | |  | |  | |  | |  | |  | |  | | .06(.15) | | -.03(.19) | | .03(.19) | | -.01(.19) |
| Mean Training Total |  | |  | |  | |  | |  | |  | |  | |  | | .01(.00) | | .01(.00) | | .01(.01) | | .01(.01) |
| Mean Barriers Total |  | |  | |  | |  | |  | |  | |  | |  | | .00(.01) | | -.01(.01) | | .00(.01) | | .01(.01) |
| Log-likelihood | -739.80 | | -912.53 | | -906.05 | | -901.42 | | -735.54 | | -902.93 | | -903.47 | | -900.09 | | -716.91 | | -884.05 | | -880.44 | | -888.15 |
| ICC  [95% CI] | .017  [.000-.491] | | .045  [.009-.203] | | .121  [.060-.230] | | .110  [.052-.217] | | .024  [.001-.331] | | .053  [.012-.198] | | .128  [.065-.237] | | .114  [.055-.222] | | .000  [.000-.000] | | .014  [.000-.652] | | .073  [.024-.199] | | .087  [.034-.202] |
| Random Effects | .09(.09) | .19(.08) | | .30(.06) | | .29(.05) | | .11(.08) | | .20(.07) | | .31(.05) | | .29(.05) | | .00(.00) | | .07(.15) | | .23(.06) | | .25(.06) | |

*Note*. Sub-scales: MHI – awareness and knowledge of mental health issues; TS = treatments and services; LP = legislation and processes; AS = comfort providing active support. * p<.05, **p<.001

Supplementary Table 8. *Multi-level models for baseline, individual-level predictors only and individual and school-level predictors for MHLCSE outcomes: complete case sensitivity analysis (N = 416, 175 schools)*

|  | Model 1: Baseline Model | | | Model 2: Individual-level Predictors | | | | | | Model 3: School-level Predictors | | | | |
| --- | --- | --- | --- | --- | --- | --- | --- | --- | --- | --- | --- | --- | --- | --- |
| Parameter Estimate | Estimate(SE) | | | Estimate(SE) | | | | | | Estimate(SE) | | | | |
|  | MHI | TS | LP | | AS | MHI | TS | LP | AS | | MHI | TS | LP | AS |
| Educator-level |  |  |  | |  |  |  |  |  | |  |  |  |  |
| Intercept | 3.56(.03)** | 2.93(.05)** | 3.15(.05)** | | 3.46(.04)** | 3.45(.08)** | 2.70(.10)** | 3.05(.11)** | 3.45(.10)** | | 3.29(.27)** | 3.14(.35)** | 2.88(.36)** | 3.19(.34)** |
| Gender (female) |  |  |  | |  | .09(.08) | .09(.11) | .06(.11) | .05(.11) | | .07(.08) | .05(.11) | .02(.11) | .04(.11) |
| Years in Practice |  |  |  | |  | .00(.00) | .01(.01)* | .00(.01) | -.00(.01) | | .00(.00) | .02(.01)* | .00(.01) | -.00(.01) |
| School-level |  |  |  | |  |  |  |  |  | |  |  |  |  |
| Designated MH Lead (yes) |  |  |  | |  |  |  |  |  | | .02(.08) | .04(.10) | .09(.11) | -.06(.10) |
| Training: selected staff only (yes) |  |  |  | |  |  |  |  |  | | -.03(.16) | -.20(.20) | -.07(.21) | -.02(.20) |
| Training: all teaching staff (yes) |  |  |  | |  |  |  |  |  | | .05(.16) | -.11(.21) | -.05(.22) | -.07(.20) |
| Mean Training Total |  |  |  | |  |  |  |  |  | | .01(.00)* | .02(.01)* | .02(.01)** | .02(.01)* |
| Mean Barriers Total |  |  |  | |  |  |  |  |  | | .00(.01) | -.02(.01) | -.00(.01) | .01(.01) |
| Log-likelihood | -419.62 | -534.61 | -533.12 | | -526.59 | -418.54 | -529.87 | -532.56 | -526.42 | | -414.55 | -523.71 | -525.14 | -522.19 |
| ICC  [95% CI] | .062  [.010-.302] | .065  [.011-.299] | .139  [.058-.299] | | .047  [.005-.338] | .064  [.011-.298] | .068  [.013-.295] | .141  [.059-.301] | .047  [.005-.340] | | .044  [.003-.384] | .039  [.002-.421] | .105  [.033-.287] | .012  [.000-.988] |
| Random Effects | .17(.08) | .22(.10) | .33(.07) | | .19(.11) | .17(.07) | .23(.09) | .33(.07) | .19(.11) | | .14(.09) | .17(.12) | .28(.08) | .09(.21) |

*Note*. Sub-scales: MHI – awareness and knowledge of mental health issues; TS = treatments and services; LP = legislation and processes; AS = comfort providing active support. * p<.05, **p<.001
