# Supplementary material for: Educators’ perceived mental health literacy and capacity to support students’ mental health: associations with school-level characteristics and provision in England
Source: Health Promot Int. 2021 Mar 1;36(6):1621–32. doi: 10.1093/heapro/daab010 (PMC8699399; doi:10.1093/heapro/daab010)
Supplement: daab010_Supplementary_Data [file daab010_supplementary_data.zip › rpSupplementary File 1..docx]

**Supplementary File 1.**

**Power Calculations**

Power was calculated for a single-level regression with 8 explanatory variables to detect a small effect (F² = .03) with standard alpha level = .05 and power = .80. This calculation indicated that a minimum sample size of 509 educators was required. Next, the design effect was calculated to account for the multi-level design using the following equation: 1 + p(m-1) where p = Intraclass Correlation Coefficient (ICC) and m = average cluster size. In the current study, the sub-scales of the MHLCSE had the following ICC values: *awareness and knowledge of mental health issues* = .017, *treatments and services* = .045, *legislation and processes* = .121, *comfort providing active support* = .110). The design effect was calculated using the average ICC across the four sub-scales (.073) and the average cluster size (2.86). The design effect was calculated at 1.14. The effective sample size (ESS) was the total sample divided by the design effect, thus 710/1.14 was equal to 623 educators. This indicates that when accounting for the multi-level design, the current study is powered to detect effects <.03.
