# Supplementary material for: Educators’ perceived mental health literacy and capacity to support students’ mental health: associations with school-level characteristics and provision in England
Source: Health Promot Int. 2021 Mar 1;36(6):1621–32. doi: 10.1093/heapro/daab010 (PMC8699399; doi:10.1093/heapro/daab010)
Supplement: daab010_Supplementary_Data [file daab010_supplementary_data.zip › rpSupplementary File 2.docx]

**Supplementary File 2.**

**Mental Health Provision Survey**

The mental health provision survey aimed to understand schools’ staffing and training relating to students’ mental health, universal and targeted mental health provision, and barriers to providing effective mental health support. The current study analysed data relating to staffing, training and perceived barriers. Specifically, ‘Does your school have a designated lead for mental health?’ (‘*yes*’/’*no’*) was coded as 0 = ‘*no designated mental health lead’* and 1 = ‘*designated mental health lead’*. A conditional item was then presented if schools responded ‘*yes*’ which asked ‘What is this mental health lead responsible for?’. Multiple responsibilities could be selected from the following options: ‘*supporting individual pupils’*, ‘*teaching pupils about mental health and wellbeing*’, ‘*training staff’*, ‘*liaising with specialist mental health services’*, ‘*coordinating and developing mental health provision in the school*’, and ‘*none of these*’.

Schools were also asked to indicate which, if any, members of staff were offered training about how to support students’ mental health and wellbeing using the following response options: ‘*all staff’*, ‘*all teaching staff*’, ‘*staff with specific responsibility for mental health*’, ‘*no staff are offered training*’ and ‘*other*’. Two mutually exclusive variables were computed. First, ‘*all staff*’ and ‘*all teaching staff*’ were combined to compute a new variable ‘*all teaching staff*’. For the purposes of this study, if all teaching staff received training, it was not important to differentiate between schools that trained all staff vs. only the teaching staff. Next, if schools did not indicate ‘*yes*’ for ‘*all teaching staff*’ the remaining responses were coded by combining both ‘*staff with specific responsibility for mental health*’ and ‘*other*’ to compute a variable for ‘*selected staff only*’. These variables were dummy coded so that a 1 indicated that the condition had been satisfied.

Schools were also asked to report, in the last two years, what staff training they had offered relating to students’ mental health and wellbeing and who provided the training. A matrix was presented with a number of different training topics down the side e.g. ‘*legislation related to young people’s mental health difficulties’* and ‘*mental health first aid*’, along with different training providers along the top e.g. ‘*voluntary organisation’* and ‘*local authority*’. Given that schools could select as many options as they wanted in the matrix, the total number of selected training options was summed to give a cumulative total score for training provision. Schools who selected no options received a score of 0, the highest possible score, if schools had selected every training topic delivered by all training providers, was 84.

Potential barriers to providing effective mental health support within school were measured using 8-items. Items were based on those previously used in the 2015, NHS England and the Department for Education Mental Health Services and Schools Link Pilots to identify the significance of potential barriers to providing effective mental health support (Day et al., 2018). Participants responded using 5-point Likert scales where 1 = ‘*very significant’*, 4 = ‘*not at all significant’* and 5 = ‘*don’t know’*, scores range from 8-32. Responses were reversed so that a high score indicated more significant barriers. Given the low number of *‘don’t know’* responses to items (0.0-6.6%), this response was coded as 1 and combined with the response *‘not at all significant’.* Cronbach’s alpha was calculated at .75 and McDonald’s omega at .77.
