# Supplementary figures and images for: Educators’ perceived mental health literacy and capacity to support students’ mental health: associations with school-level characteristics and provision in England
Source: Health Promot Int. 2021 Mar 1;36(6):1621–32. doi: 10.1093/heapro/daab010 (PMC8699399; doi:10.1093/heapro/daab010)

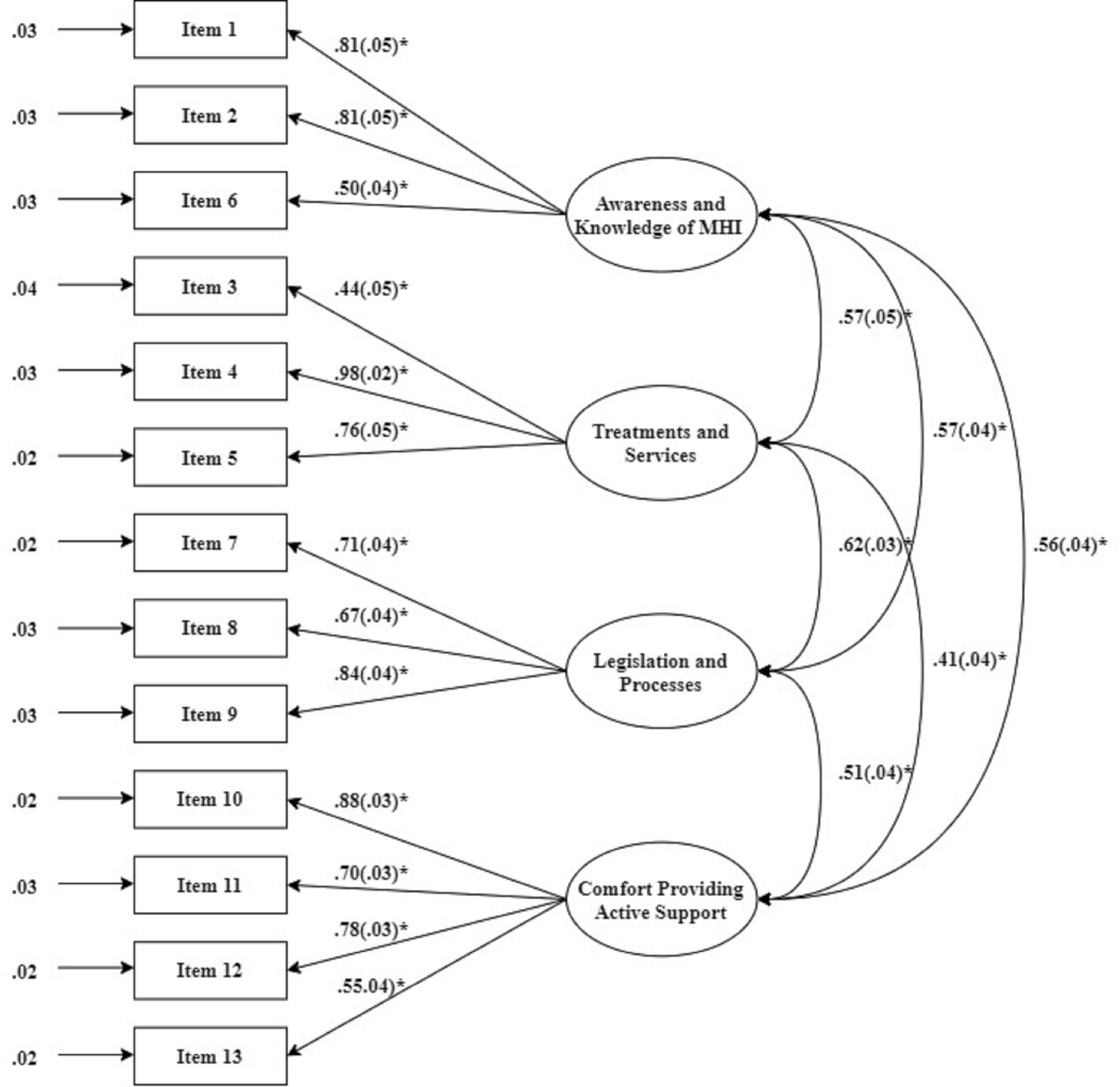

Supplement: daab010_Supplementary_Data [file daab010_supplementary_data.zip › rpSupplementary Figure.tif]
